# Na^+^-Intercalated Two-Dimensional Vermiculite-Modified TiO_2_ Electron Transport Layer for Enhanced CsPbI_2_Br Perovskite Solar Cells

**DOI:** 10.3390/nano16191216

**Published:** 2026-09-26

**Authors:** Yuan Xu, Yaxuan Chai, Kaituo Zhang, Fengli Liu, Tiantian Li, Ke Xu, Xian Hua, Pengju Guo, Fachuang Li, Lan Zhang

**Affiliations:** 1 Xinxiang Engineering Research Center for Advanced Energy Storage Batteries and Materials, School of Materials Science and Engineering, Henan Institute of Technology, Xinxiang 453003, China; 2 Henan Key Laboratory of Advanced Cable Materials and Intelligent Manufacturing, School of Cable Engineering, Henan Institute of Technology, Xinxiang 453003, China; 3 Fujian Key Laboratory of Photoelectric Functional Materials, College of Materials Science & Engineering, Huaqiao University, Xiamen 361021, China; 4 Engineering Technology Education Center, Henan Institute of Technology, Xinxiang 453003, China

**Keywords:** all-inorganic perovskite solar cells, CsPbI_2_Br, TiO_2_ electron transport layer, vermiculite nanosheets, interface engineering

## Abstract

Interface engineering of the electron transport layer (ETL) is an effective strategy for enhancing the performance and stability of all-inorganic perovskite solar cells (PSCs). Herein, Na^+^-intercalated vermiculite nanosheets were introduced as a low-cost natural interfacial modifier for the TiO_2_ ETL in CsPbI_2_Br PSCs. The nanosheets were prepared via an aqueous-phase exfoliation process and deposited onto the TiO_2_ surface to regulate the buried TiO_2_/CsPbI_2_Br interface. Morphological and spectroscopic characterizations revealed that vermiculite modification reduced the surface roughness of TiO_2_, altered its surface chemical/electronic environment and was associated with improved interfacial energy-level alignment. As a result, the quality of the CsPbI_2_Br film was significantly improved, leading to larger grains, enhanced crystallinity, a lower apparent trap-state density, and more efficient interfacial charge-transfer behavior. Electrical characterizations further confirmed enhanced charge extraction, reduced carrier recombination, and an increased built-in potential in the modified devices. Consequently, the vermiculite-modified devices exhibited simultaneous improvements in power conversion efficiency (PCE), open-circuit voltage, and short-circuit current density, with the best-performing device reaching a PCE of 13.94%. In addition, the modified device retained 84.51% of its initial PCE after storage in air for 35 days, significantly higher than the 71.1% retained by the control device. These findings highlight the potential of Na^+^-intercalated vermiculite nanosheets as low-cost natural modifiers for TiO_2_/CsPbI_2_Br buried-interface engineering in efficient and stable all-inorganic PSCs.

## 1. Introduction

Lead halide perovskites are considered highly promising photovoltaic materials owing to their excellent optoelectronic properties, including long carrier diffusion lengths, solution processability, tunable bandgaps, high carrier mobility, and strong light absorption coefficients [1,2,3,4]. In recent years, perovskite solar cells (PSCs) have developed rapidly. The certified power conversion efficiency (PCE) of single-junction organic–inorganic hybrid PSCs has reached 27.3%, while the highest reported efficiency of all-inorganic CsPbI_2_Br PSCs has reached 17.34% [5,6]. The electron transport layer (ETL) plays a crucial role in achieving high-performance PSCs [7,8]. Among transition-metal oxides, various materials such as Al_2_O_3_, ZnO, TiO_2_, and SnO_2_ have been widely employed as ETLs to improve device efficiency [9,10,11]. Among them, TiO_2_ is one of the most commonly used ETLs because of its wide bandgap, long electron lifetime, low cost, facile fabrication, and suitable band alignment relative to other electron transport materials [12]. In addition, the conduction band of TiO_2_ is favorably aligned with that of the perovskite absorber, which facilitates electron transfer from the perovskite layer to the compact TiO_2_ layer [13]. However, TiO_2_ still suffers from several drawbacks, including relatively low electron mobility, poor conductivity, and possible photocatalytic degradation under ultraviolet irradiation [14]. Therefore, it is necessary to improve the conductivity and electron transport capability of TiO_2_ through interfacial engineering or material modification, while simultaneously suppressing its photocatalytic activity and optimizing the energy-level alignment at the ETL/perovskite interface, thereby promoting electron extraction and reducing interfacial recombination [15].

Two-dimensional (2D) materials, such as MoS_2_, MXenes, graphene, and 2D perovskites, possess unique advantages including atomic-scale thickness, high carrier mobility, abundant surface functional groups, tunable energy bands, and good chemical stability [16,17,18,19,20]. These characteristics enable them to improve perovskite film quality and device performance through interfacial passivation, grain-boundary engineering, and band-structure modulation [21,22,23,24,25]. Moreover, surface-functionalized 2D materials can reduce the interfacial barrier between the ETL and perovskite layer, passivate high-density defects, and suppress interfacial charge recombination [26]. Nevertheless, many representative 2D materials, such as MXenes, remain expensive, difficult to store, complicated to prepare, and unstable in air. Therefore, it is particularly important to explore low-cost and readily available 2D materials for buried-interface modification toward efficient and stable PSCs [27]. In addition to inorganic and two-dimensional interfacial modifiers, molecular interlayers have also emerged as an effective strategy for buried-interface engineering in PSCs. Such interlayers can regulate interfacial electronic coupling, tune local energy-level alignment, and reduce interfacial recombination losses, thereby facilitating charge extraction and improving device performance. Recent studies have shown that carefully designed molecular interlayers can produce loss-reduced interfaces even without changing the overall device architecture [28]. These results further highlight that interfacial chemistry and electronic coupling are central factors in PSC optimization. Compared with these molecular interlayers, natural layered silicates such as vermiculite provide a materially distinct but conceptually related route for interface regulation, owing to their lamellar structure, ion-exchangeable interlayers, and thermal robustness.

In this regard, naturally abundant layered minerals, such as vermiculite, kaolinite, and talc, are attractive because of their low cost, wide availability, and relatively environmentally friendly processing. In particular, layered natural minerals can be exfoliated into 2D nanosheets and thus hold great promise as low-cost interfacial materials for PSCs. For example, Hsin et al. employed montmorillonite–CH_3_NH_3_PbI_3_ nanocomposites as photosensitive layers and achieved stable inverted PSCs with a PCE of 17.29% [29]. Recently, Zhang et al. reported alkali-intercalated vermiculite as a buried-interface modifier for carbon-based inorganic perovskite solar cells and demonstrated its effectiveness in improving both CsPbIBr_2_ and CsPbI_2_Br devices, with a CsPbI_2_Br power conversion efficiency reaching 15.25% [30]. Their study further compared different intercalated alkali ions and mainly attributed the performance enhancement to template-assisted crystal growth and alkali-ion-related passivation/diffusion effects. This work therefore represents a close and important precedent for the present study. In view of this, the present work is not intended as the first demonstration of alkali-intercalated vermiculite in perovskite solar cells. Instead, we focus specifically on the Na^+^-intercalated vermiculite/TiO_2_/CsPbI_2_Br buried interface and investigate, in a more interface-centered manner, how the modifier regulates the TiO_2_ electron transport layer, including its surface morphology, surface potential, chemical environment, and energy-level alignment, and how these interfacial changes correlate with subsequent CsPbI_2_Br film growth and charge-extraction behavior for Table S1.

In this work, Na^+^-intercalated expanded vermiculite was used to modify the TiO_2_ ETL in CsPbI_2_Br PSCs. Because of its layered structure, ion-exchangeable interlayers, and good thermal robustness after expansion, expanded vermiculite is a suitable natural precursor for intercalation-assisted exfoliation and buried-interface modification in all-inorganic PSCs. Here, Na^+^-intercalated vermiculite nanosheets were prepared via a water-phase exfoliation process and introduced onto the TiO_2_ ETL to regulate the buried TiO_2_/CsPbI_2_Br interface. In particular, this work emphasizes how the modifier affects the TiO_2_-side interfacial properties, including surface morphology, surface potential, chemical environment, and energy-level alignment, and how these changes are associated with perovskite film growth and interfacial charge transport. As a result, the optimized device achieved a PCE of 13.94% and retained 84.51% of its initial efficiency after storage in air for 35 days.

## 2. Experimental Section

### 2.1. Materials

Fluorine-doped tin oxide (FTO) glass substrates (14 Ω sq^−1^), lead iodide (PbI_2_, 99.99%), lead bromide (PbBr_2_, 99.99%) and cesium iodide (CsI, 99.99%) were purchased from Liaoning Youxuan New Energy Technology Co., Ltd. (Huludao, China). Titanium tetrachloride (TiCl_4_, 99%) was obtained from Macklin (Shanghai Macklin Biochemical Co., Ltd., Shanghai, China). Dimethyl sulfoxide (DMSO, AR, 99.7%), N,N-dimethylformamide (DMF, AR, 99.5%), isopropanol (AR), acetonitrile (AR), and absolute ethanol (AR) were purchased from Sinopharm Chemical Reagent Co., Ltd. (Shanghai, China) and used as received. Conductive carbon paste was purchased from Guangzhou Seaside Technology Co., Ltd. (Guangzhou, China). Vermiculite (85%) was purchased from Shanghai Yuanye Bio-Technology Co., Ltd. (Shanghai, China). Sodium chloride (98%) was obtained from Sinopharm Chemical Reagent Co., Ltd. (Shanghai, China). Both were used without further purification. All chemicals were used as received unless otherwise stated.

### 2.2. Synthesis of Vermiculite Nanosheets

#### Synthesis of Vermiculite Intercalation Compound (Vic)

Natural vermiculite powder was dispersed in 3.4 M NaCl aqueous solution and treated at 180 °C for 12 h to promote Na^+^-assisted ion exchange/intercalation. After the reaction, the solid product was collected by centrifugation and washed repeatedly with deionized water to remove residual soluble salts. The washing process involved repeated redispersion and centrifugation cycles until the supernatant was free of obvious residual salt. The purified Na^+^-treated vermiculite was then redispersed in deionized water and exfoliated by ultrasonication. The resulting supernatant was collected and filtered to remove large aggregates, yielding a stable vermiculite nanosheet dispersion for subsequent modification of the TiO_2_ electron transport layer.

It should be noted that the present study focuses on the interfacial effect of the deposited Na^+^-intercalated vermiculite nanosheet layer as a whole. Because untreated bulk vermiculite is difficult to process into a comparable uniform interfacial coating under the same conditions, the individual contributions of Na^+^ intercalation and exfoliated-sheet morphology are not fully decoupled here.

### 2.3. Device Fabrication

#### 2.3.1. Fto Substrate Cleaning and Patterning

The FTO substrates were patterned by laser etching to define the bottom electrode, and the etched region was approximately 3 mm × 15 mm. The patterned substrates were cleaned with detergent and deionized water, followed by sequential ultrasonic cleaning in deionized water and absolute ethanol. After cleaning, the substrates were dried in an oven at 60 °C.

#### 2.3.2. TiO_2_ Electron Transport Layer

Compact TiO_2_ films were prepared by a chemical bath deposition method. Briefly, 4.5 mL of TiCl_4_ was slowly added dropwise into 200 mL of ice-cooled deionized water under stirring. After complete melting of the ice, a transparent precursor solution was obtained. The cleaned FTO substrates were treated with UV–ozone for 30 min and then immersed in 40 mL of the TiCl_4_ precursor solution. The solution was kept at 70 °C for approximately 24 min until it turned bright blue. The deposited substrates were then removed, thoroughly rinsed with deionized water and absolute ethanol, spin-dried, and annealed at 200 °C for 1 h in air. After cooling to room temperature, the TiO_2_-coated substrates were treated again with UV–ozone before further use.

#### 2.3.3. Vermiculite Modification of TiO_2_

For vermiculite modification, 60 μL of the purified Na^+^-treated vermiculite nanosheet dispersion was deposited onto the TiO_2_-coated FTO substrate by spin coating at 4000 rpm, followed by annealing at 150 °C for 10 min. The unmodified TiO_2_ substrate was used as the control.

#### 2.3.4. CsPbI_2_Br Perovskite Absorber

The CsPbI_2_Br precursor solution was prepared in a nitrogen-filled glovebox by dissolving 0.3118 g CsI, 0.2766 g PbI_2_, and 0.2202 g PbBr_2_ in 1 mL DMSO, corresponding to a 1.2 M precursor solution. The precursor was stirred until completely dissolved and filtered through a 0.22 μm organic syringe filter before use.

Before perovskite deposition, the TiO_2_ or TiO_2_/vermiculite substrates were treated by UV–ozone for 15 min and plasma cleaning for 5 min, followed by preheating at 60 °C for 10 min. Then, 50 μL of the precursor solution was spin-coated onto the substrate using a two-step program of 700 rpm for 5 s and 3500 rpm for 35 s. After spin coating, the films were first heated at 38 °C for 80 s and then annealed at 180 °C for 10 min.

#### 2.3.5. Carbon Electrode

After formation of the perovskite film, the edge of the conductive substrate was wiped with a cotton swab wetted with DMF to expose the FTO contact region (approximately 1 mm × 15 mm). Commercial conductive carbon paste was then deposited on top of the perovskite layer by screen printing, followed by annealing at 120 °C for 15 min to complete the device fabrication.

#### 2.3.6. Device Active Area

The active area of the devices was 0.04 cm^2^, defined by the overlap area between the FTO bottom electrode and the carbon top electrode. A metal shadow mask with the same aperture area was used during photovoltaic measurements.

### 2.4. Characterization

The morphology, composition, electronic structure, optical properties, and photovoltaic performance of the samples were characterized by SEM, XRD, AFM, KPFM, XPS, UPS, UV–vis spectroscopy, steady-state PL, TRPL, J–V, EQE, EIS, Mott–Schottky, TPV/TPC, and SCLC measurements. Detailed instrument models and measurement conditions are provided in the Supporting Information.

## 3. Results and Discussion

### 3.1. Effect of Intercalated Vermiculite on the TiO_2_ ETL

Figure 1a shows the SEM image of the intercalated vermiculite, revealing a typical stacked sheet-like morphology. The lateral size of the intercalated vermiculite nanosheets is approximately 1.2 μm. Although the lateral dimensions are at the micrometer scale, the nanosheets can still be relatively uniformly distributed on the TiO_2_ surface. The TEM images in Figure 1b,c further confirm the two-dimensional sheet-like structure, with a thickness of approximately 10 nm and a typical layered stacking feature. Such a 2D layered structure is expected to provide continuous pathways for interfacial charge transport and help reduce charge accumulation induced by interfacial defects.

Figure 1d,e present the SEM images of TiO_2_ films before and after vermiculite modification, respectively. Compared with bare TiO_2_, the modified TiO_2_ surface exhibits a more uniform and compact morphology, while the overall particle morphology changes only slightly. This indicates that the vermiculite nanosheets are mainly distributed on the TiO_2_ surface rather than embedded within the TiO_2_ layer, which helps construct a flatter and less defective interfacial layer and provides a more favorable substrate for subsequent perovskite growth.

Atomic force microscopy (AFM) was further employed to investigate the surface morphology of the TiO_2_ films. As shown in Figure S1a,c, the arithmetic average roughness (Ra) of the TiO_2_ films decreases from 22.1 nm to 17.2 nm after vermiculite modification, while the root mean square roughness (Rq) decreases from 27.4 nm to 21.62 nm. The reduced surface roughness suggests that the vermiculite nanosheets partially cover surface asperities and locally fill or bridge uneven regions on the TiO_2_ film, leading to a more uniform effective buried interface rather than implying that the nanosheets themselves are intrinsically smoother than TiO_2_. Such interfacial planarization is expected to improve interfacial contact and benefit charge extraction and the fill factor of the device.

Kelvin probe force microscopy (KPFM) was used to probe the surface potential variation in the TiO_2_ ETL before and after modification. As shown in Figure S1b,d, the TiO_2_/vermiculite film exhibits a higher average surface potential than the bare TiO_2_ film, indicating that vermiculite modification changes the surface electronic environment of the ETL and may be favorable for interfacial energy-level alignment with the perovskite layer.

To further investigate the electronic interactions induced by vermiculite modification, X-ray photoelectron spectroscopy (XPS) measurements were carried out to examine the changes in the binding energies of surface elements. As shown in Figure 2a, a distinct Na 1s peak appears in the XPS spectrum of the vermiculite-modified TiO_2_ film, indicating that Na^+^-intercalated vermiculite was successfully introduced onto the TiO_2_ surface.

Figure 2b,c show the Ti 2p and O 1s spectra of TiO_2_ films before and after modification. The O 1s spectrum can be deconvoluted into three components, which are assigned to lattice oxygen, defect-related/non-lattice oxygen, and chemically adsorbed oxygen species, consistent with previous reports on TiO_2_-based oxide surfaces [31]. For the control sample, these peaks are located at 530.26, 531.71, and 533.22 eV, respectively, while after vermiculite modification, they shift to lower binding energies of 529.90, 531.23, and 532.52 eV. This is consistent with a change in the surface chemical/electronic environment of oxygen species on TiO_2_ after vermiculite modification. However, because XPS binding-energy shifts can also be influenced by differential sample charging, the observed shifts are interpreted here more cautiously as evidence of a modified interfacial chemical/electronic environment rather than as direct proof of charge transfer alone. After vermiculite treatment, the intensity of the lattice oxygen peak decreases, whereas the intensity of the chemically adsorbed oxygen peak increases. In addition, enhanced oxygen-related surface species are observed at 531.23 and 532.51 eV, corresponding to defect-related oxygen and adsorbed oxygen, respectively. These results suggest that vermiculite modification alters the surface oxygen chemistry of TiO_2_ and is accompanied by an increased contribution from oxygen-containing surface species. Meanwhile, the Ti 2p peaks shift from 458.85 and 464.53 eV to 458.49 and 464.18 eV, respectively. Because such small binding-energy shifts may also be affected by differential charging, they are not taken here as standalone proof, but rather considered together with the AFM, KPFM, UPS, and device-level measurements, which collectively support a modified buried interfacial environment after vermiculite treatment. These findings indicate that vermiculite modification changes the surface chemical environment of TiO_2_ and may influence oxygen-vacancy-related surface states or other TiO_2_-side interfacial states. Such changes are expected to affect interfacial charge-transfer behavior and may contribute to mitigating defect-assisted recombination. More importantly, these TiO_2_-side changes support the view that the role of Na^+^-intercalated vermiculite in the present system is not limited to perovskite crystal-growth modulation, but also involves regulation of the ETL surface and buried interfacial energy landscape.

Figure 2d shows the XRD patterns of perovskite films deposited on TiO_2_ before and after vermiculite modification. The diffraction peaks corresponding to the (200) and (100) planes are located at 29.3° and 14.4°, respectively. The modified perovskite film shows enhanced diffraction intensity, indicating improved crystallinity and enlarged grain size of the CsPbI_2_Br film, without generating any detectable impurity phase.

### 3.2. Composition and Morphology Analysis of the Perovskite Films

Interfacial modification of the ETL can regulate the buried-interface properties and thereby influence the nucleation and crystallization behavior of the perovskite layer, leading to improved photovoltaic performance of CsPbI_2_Br devices. To evaluate the effect of vermiculite-modified TiO_2_ on the crystal structure and surface morphology of perovskite films, top-view SEM images were collected. As shown in Figure 3a,d, compared with the control sample, the CsPbI_2_Br film grown on the vermiculite-modified TiO_2_ substrate exhibits larger grains and fewer grain boundaries. This observation is consistent with the AFM results, indicating that the CsPbI_2_Br film grown on the TiO_2_/vermiculite substrate is smoother and composed of larger grains, suggesting that the vermiculite-modified buried interface effectively regulates perovskite film growth. A more uniform and compact perovskite film is beneficial for reducing grain-boundary defects and interfacial recombination, thereby improving short-circuit current density (J_SC_) and fill factor (FF). In the present case, however, the improved film growth should be understood as a consequence of TiO_2_-side buried-interface regulation rather than as an isolated crystal-growth effect.

The grain-size distribution histograms in Figure 3b,e further demonstrate that the average grain size of the CsPbI_2_Br film increases from 0.42 to 0.70 μm after vermiculite modification, and the maximum grain size reaches 1.18 μm. The more compact grain arrangement in the modified film is expected to suppress non-radiative recombination at grain boundaries and facilitate charge transport. Cross-sectional SEM images of the devices (Figure 3c,f) further reveal that the perovskite film deposited on the vermiculite-modified ETL has higher film quality and more intimate contact with the carbon electrode.

To further clarify the influence of vermiculite modification on the surface chemical environment of CsPbI_2_Br films, XPS measurements were performed. As shown in Figure S2, the XPS spectra of CsPbI_2_Br films deposited on pristine and vermiculite-modified TiO_2_ were compared. In Figure S2d, the Br 3d5/2 and Br 3d3/2 peaks of the control CsPbI_2_Br film are located at 69.14 and 68.06 eV, respectively. After vermiculite modification, these peaks shift to 68.60 and 67.51 eV, respectively, showing an overall shift toward lower binding energy. Meanwhile, the Pb 4f, I 3d, and Cs 3d peaks also exhibit similar shifts. These results indicate that vermiculite modification changes the local chemical environment of the perovskite surface, which may reflect a redistribution of interfacial electron density and possible changes in defect-related surface states.

### 3.3. Carrier Dynamics of the Perovskite Films

Steady-state photoluminescence (PL) and time-resolved photoluminescence (TRPL) measurements were first carried out to investigate the carrier-transfer dynamics. A 450 nm excitation wavelength was used for the PL measurements. As shown in Figure S3a, the characteristic emission peak of the CsPbI_2_Br films remains at 650 nm before and after vermiculite modification, indicating that the modification has a negligible effect on the perovskite composition. However, the PL intensity of the CsPbI_2_Br film is markedly reduced after vermiculite modification, suggesting more efficient transfer of photogenerated carriers from the perovskite layer to the TiO_2_ ETL.

Figure S3b shows the TRPL spectra of CsPbI_2_Br films before and after treatment with intercalated vermiculite. The TRPL decay curves were fitted using a biexponential decay function to extract the parameters associated with defect-assisted non-radiative recombination and radiative recombination, and the average carrier lifetime (τ_ave_) was calculated. The detailed fitting parameters are summarized in Table S2. The fitted TRPL results reveal that the perovskite film deposited on TiO_2_/vermiculite exhibits a shorter average decay lifetime, decreasing from 23.98 ns to 9.05 ns. This behavior is consistent with faster interfacial charge extraction and more efficient carrier transfer from CsPbI_2_Br to the TiO_2_ ETL, rather than serving as direct evidence for reduced bulk non-radiative recombination within the perovskite.

To further investigate the optical properties of TiO_2_ films with and without vermiculite modification, the optical transmittance spectra of FTO/TiO_2_ and FTO/TiO_2_/vermiculite were measured. As shown in Figure 4a, vermiculite modification enhances the transmittance of the TiO_2_ film in the wavelength range of 400–500 nm. The corresponding Tauc plots (Figure 4b) show that the optical bandgaps (E_g_) of TiO_2_ and TiO_2_/vermiculite are 3.89 and 3.85 eV, respectively. The Tauc analysis indicates that the optical bandgap of TiO_2_ decreases slightly after vermiculite modification. Combined with the UPS results, this suggests a slight downward shift in the conduction-band position, which optimizes the energy-level alignment between the ETL and CsPbI_2_Br, reduces the interfacial barrier for electron extraction, and suppresses interfacial recombination. Together with the AFM, KPFM, and XPS results, these observations support an interface-centered mechanism in which Na^+^-intercalated vermiculite modifies the TiO_2_-side buried junction electronically and chemically, beyond merely assisting perovskite crystallization. This change is consistent with the higher open-circuit voltage (V_OC_) and improved charge-transport behavior observed in the corresponding devices.

Since the buried interface strongly affects the crystallization of the perovskite absorber, AFM and KPFM measurements were further performed to investigate the surface electronic characteristics of the optimized CsPbI_2_Br films. As shown in Figure 5a,d, the arithmetic average roughness (Ra) of the control and optimized perovskite films decreases from 11.3 nm to 6.98 nm, while the root mean square roughness (Rq) decreases from 13.9 nm to 8.87 nm. These results indicate that vermiculite treatment significantly reduces the roughness of the CsPbI_2_Br film and renders the film smoother, in good agreement with the SEM observations. This improvement is attributed to the regulation of the buried TiO_2_/CsPbI_2_Br interface, where the layered vermiculite nanosheets can partially level local surface irregularities and modify the interfacial wettability/energy, thereby affecting precursor spreading, nucleation, and subsequent crystal growth. As a result, the perovskite film becomes more uniform, which is favorable for tighter contact with the carbon electrode and for charge transport and extraction.

The contact potential difference (CPD) of the perovskite films was further analyzed by KPFM. As shown in Figure 5b,e, the optimized perovskite film exhibits a higher surface potential and a narrower potential distribution than the control film. This suggests that the optimized film is more uniform and that local charge accumulation induced by defect-related under-coordinated Pb species is suppressed. In addition, water contact angle measurements were conducted to evaluate the wettability of the perovskite films. As shown in Figure 5c,f, the contact angles of the CsPbI_2_Br films deposited on TiO_2_ and TiO_2_/vermiculite are 37.21° and 54.54°, respectively. The increased contact angle indicates enhanced hydrophobicity, which helps suppress moisture penetration and improve the environmental stability of the devices. Nevertheless, the long-term stability remains limited by other degradation pathways in the carbon-based CsPbI_2_Br device stack, such as residual interfacial defects, ion migration, and gradual perovskite/interface degradation under ambient exposure.

To better understand the effect of vermiculite on charge dynamics and energy-level alignment at the buried interface, ultraviolet photoelectron spectroscopy (UPS) was employed to investigate the band structures of the relevant components. First, UV–vis absorption spectra were measured for TiO_2_/CsPbI_2_Br and TiO_2_/vermiculite/CsPbI_2_Br. The absorption spectra and Tauc plots shown in Figure 6a,b indicate that the bandgaps of the control and modified perovskite films are 1.92 and 1.91 eV, respectively. The nearly unchanged bandgap suggests that vermiculite modification does not significantly alter the intrinsic optical properties of CsPbI_2_Br, and that the improved device performance mainly originates from optimized interfacial energy alignment and defect suppression.

UPS measurements were then conducted for TiO_2_, TiO_2_/vermiculite, and CsPbI_2_Br to determine their energy-level structures. The conduction-band minimum (CBM) of the ETL was estimated by combining the UPS-derived valence-band maximum (VBM) with the optical bandgap obtained from UV–vis absorption analysis. Enlarged UPS spectra of the valence-band onset (E_onset_) and secondary electron cutoff (E_cutoff_) are shown in Figure 6c–e. The value of VBM can be calculated according to the equation of $VBM=21.22\;\mathrm{eV}-(E_{cutoff}-E_{onset})$. For the TiO_2_ film, E_cutoff_ and E_onset_ are 16.62 and 2.76 eV, respectively, giving calculated VBM and CBM values of −7.36 and −3.47 eV. For the TiO_2_/vermiculite film, the corresponding VBM and CBM values are −7.41 and −3.56 eV, respectively. The detailed parameters are summarized in Table S3. The resulting energy-level alignment of the ETL/perovskite interface is illustrated in Figure 6f. These results suggest that vermiculite modification slightly shifts the ETL energy levels and leads to a more favorable interfacial energy-level alignment with the perovskite absorber, which is consistent with more efficient electron extraction and the improved V_OC_ and device performance. It should be noted that the optical bandgap values extracted for the TiO_2_-based thin films are higher than those typically reported for bulk anatase TiO_2_. This discrepancy may arise from the thin-film absorption analysis, uncertainty associated with the Tauc fitting of weak absorption-edge features, and the limited fitting range. Therefore, in the present work these values are mainly used to estimate the relative shift in band-edge positions after vermiculite modification, rather than to establish absolute TiO_2_ bandgap values.

### 3.4. Photovoltaic Performance and Stability of the Perovskite Devices

After clarifying the interfacial energy-level regulation induced by vermiculite, electrical characterizations were further conducted to elucidate its influence on charge transport and recombination in the devices. Electrochemical impedance spectroscopy (EIS) was employed to analyze the carrier dynamic processes and provide insight into photogenerated charge transport in the perovskite layer. Figure 7a presents the Nyquist plots measured in the dark, and the fitted parameters are summarized in Table S4. By fitting the spectra with an equivalent circuit, the series resistance (R_s_) and recombination resistance (R_rec_) were obtained. The vermiculite-modified PSC exhibits an R_rec_ value of 22,476 Ω, which is much higher than that of the unmodified device (9023 Ω), indicating that charge recombination is significantly suppressed in the modified PSCs. Reduced recombination is beneficial for carrier extraction and is one of the main reasons for the improved device performance.

To further evaluate the effect of vermiculite modification on charge recombination, Mott–Schottky (M–S) measurements were conducted, as shown in Figure 7b. After modification, the extracted built-in potential (V_bi_) increases from 1.05 to 1.22 V, indicating a stronger driving force for charge separation and transport, which is beneficial for improving V_OC_. Transient photovoltage (TPV) and transient photocurrent (TPC) measurements were also performed. TPV provides insight into the carrier decay process in PSCs. As shown in Figure 7c, the photovoltage decay lifetime is prolonged from 1.77 to 22.97 ms after vermiculite modification, indicating slower voltage decay, longer carrier lifetime, and suppressed recombination. In contrast, the modified device exhibits enhanced carrier extraction and transport in the TPC measurement. As shown in Figure 7d, the photocurrent decay lifetime decreases from 20.30 to 12.50 μs, indicating a shorter charge-extraction time and faster current response. Faster photocurrent decay is generally associated with more efficient charge transfer, confirming the improved charge extraction and transport in the modified device.

The dependences of V_OC_ and J_SC_ on light intensity were further measured to reveal the recombination mechanism. The ideality factor (N) was determined from the slope of V_OC_ versus light intensity. According to the V_OC_–light intensity plots shown in Figure S4b, the N value decreases from 1.12 kT/q for the control device to 0.99 kT/q for the vermiculite-modified device. A N value closer to 1 indicates weaker trap-assisted recombination. The J_SC_ dependence on light intensity is shown in Figure S4a. The fitted slope (α) decreases from 1.17 for the control device to 1.07 for the modified device. In principle, α values close to unity indicate weak bimolecular recombination. Here, the slightly superlinear α values may arise from experimental uncertainty in light-intensity calibration, fitting error, or small deviations in current collection behavior under different illumination intensities. Because the deviation from unity is limited, we do not interpret this result as evidence of a distinct superlinear physical mechanism. Overall, the α values remain close to 1, consistent with relatively weak bimolecular recombination, and the lower α value of the modified device is in line with the EIS, TPV, and TPC analyses.

To evaluate the influence of vermiculite modification on the photovoltaic performance of CsPbI_2_Br PSCs, J–V measurements were carried out, and the corresponding curves are shown in Figure 8a. The detailed photovoltaic parameters are summarized in Table S5. As expected, both V_OC_ and J_SC_ are significantly improved after vermiculite modification. The modified device achieves a champion PCE of 13.94%, with a J_SC_ of 14.44 mA·cm^−2^, an FF of 77.60%, and a V_OC_ of 1.24 V. In contrast, the control device exhibits a PCE of only 12.25%, with a J_SC_ of 13.50 mA·cm^−2^, an FF of 76.80%, and a V_OC_ of 1.18 V. To further evaluate scan-direction dependence, forward and reverse J–V curves were measured (Figure S5), and the hysteresis index was calculated (Table S6). The vermiculite-modified device exhibits reduced hysteresis compared with the control, consistent with improved interfacial charge extraction and reduced interfacial trapping. In addition, a NaCl-only control was examined to evaluate whether the observed improvement could arise simply from residual soluble sodium salts. As shown in Figure S6, the NaCl-only treatment does not reproduce the full enhancement in J-V characteristics and PL quenching observed for the Na^+^-intercalated vermiculite nanosheets, indicating that the dominant device improvement does not arise from residual NaCl alone, although a minor contribution from residual soluble ions cannot be completely excluded.

External quantum efficiency (EQE) and steady-state output measurements were further conducted to evaluate the integrated current density and output stability of the devices. The corresponding results are shown in Figure 8b,d. As shown in Figure 8b, the integrated current densities derived from the EQE spectra are 13.52 and 14.20 mA·cm^−2^ for the control and modified devices, respectively, which are in good agreement with the J_SC_ values obtained from the J–V measurements. The enhanced EQE can be attributed to the improved crystalline quality of the CsPbI_2_Br film and reduced non-radiative recombination after vermiculite modification. To further evaluate the trap-state density in the perovskite films, the space-charge-limited current (SCLC) method was applied to devices with an FTO/TiO_2_/perovskite/carbon electrode structure. As shown in Figure 8c, the trap-filled limit voltage (V_TFL_) is 1.11 V for the control device and 0.98 V for the vermiculite-modified device. The trap density (N_t_) was calculated accordingly. After vermiculite treatment, the N_t_ value of the CsPbI_2_Br film decreases from 8.07 × 10^15^ cm^−3^ to 6.47 × 10^15^ cm^−3^. The reduced trap-state density suggests that vermiculite modification suppresses defect formation and improves the crystallization quality of the perovskite film. It should be noted that the FTO/TiO_2_/perovskite/carbon structure used here is not an ideal single-carrier-only SCLC device. Therefore, the extracted trap-density values are interpreted mainly as apparent/comparative indicators of trap-related charge-transport behavior under the same device architecture, rather than as absolute bulk trap densities. Figure 8d shows the steady-state output at the maximum power point. After 400 s of continuous operation, the stabilized PCEs reach 12.3% and 13.77% for the control and modified devices, respectively, which are within an acceptable deviation from the values obtained from the J–V curves, further confirming the reliability of the photovoltaic parameters.

In addition, to more intuitively evaluate device reproducibility, 15 devices were tested and the box plots of PCE, V_OC_, FF, and J_SC_ were statistically analyzed, as shown in Figure S7. The vermiculite-modified devices exhibit a narrower PCE distribution and better reproducibility, which is closely related to their higher and more concentrated V_OC_ and J_SC_ values. The significantly improved V_OC_ and J_SC_ can be attributed to the better interfacial contact between TiO_2_ and the CsPbI_2_Br film after vermiculite modification, as well as the suppression of non-radiative recombination.

The long-term stability of the devices was evaluated by storing them under ambient conditions at a relative humidity of approximately 25% and a temperature of 24 °C for 35 days (840 h), as shown in Figure S8. The vermiculite-modified CsPbI_2_Br PSC retains 84.51% of its initial PCE after 35 days, whereas the control device retains only 71.1%. These results indicate that vermiculite modification significantly improves the environmental stability of the device, which can be mainly attributed to passivation of ETL/perovskite interfacial defects, improved perovskite film quality, and enhanced hydrophobicity of the film.

## 4. Conclusions

In summary, Na^+^-intercalated vermiculite nanosheets were introduced to modify the TiO_2_ ETL in CsPbI_2_Br PSCs. The buried interfacial modification optimized the energy-level alignment and surface chemical environment at the TiO_2_/CsPbI_2_Br interface, promoted perovskite crystallization, and likely reduced interfacial loss sites. As a result, interfacial charge extraction and transport were enhanced, while non-radiative recombination losses were mitigated. The vermiculite-modified device exhibited simultaneously improved V_OC_ and J_SC_, delivering a champion PCE of 13.94%. Meanwhile, the modified CsPbI_2_Br film showed a denser morphology and enhanced hydrophobicity, enabling the device to retain 84.51% of its initial PCE after storage in air for 35 days. These results demonstrate that Na^+^-intercalated vermiculite nanosheets can serve as promising low-cost natural interfacial modifiers for carbon-based efficient and stable all-inorganic perovskite solar cells. More importantly, the present study provides an interface-centered understanding of how Na^+^-intercalated vermiculite regulates the TiO_2_-side buried junction in CsPbI_2_Br devices, thereby distinguishing the present work from a simple cation or composition variation.

## Figures and Tables

**Figure 1:**
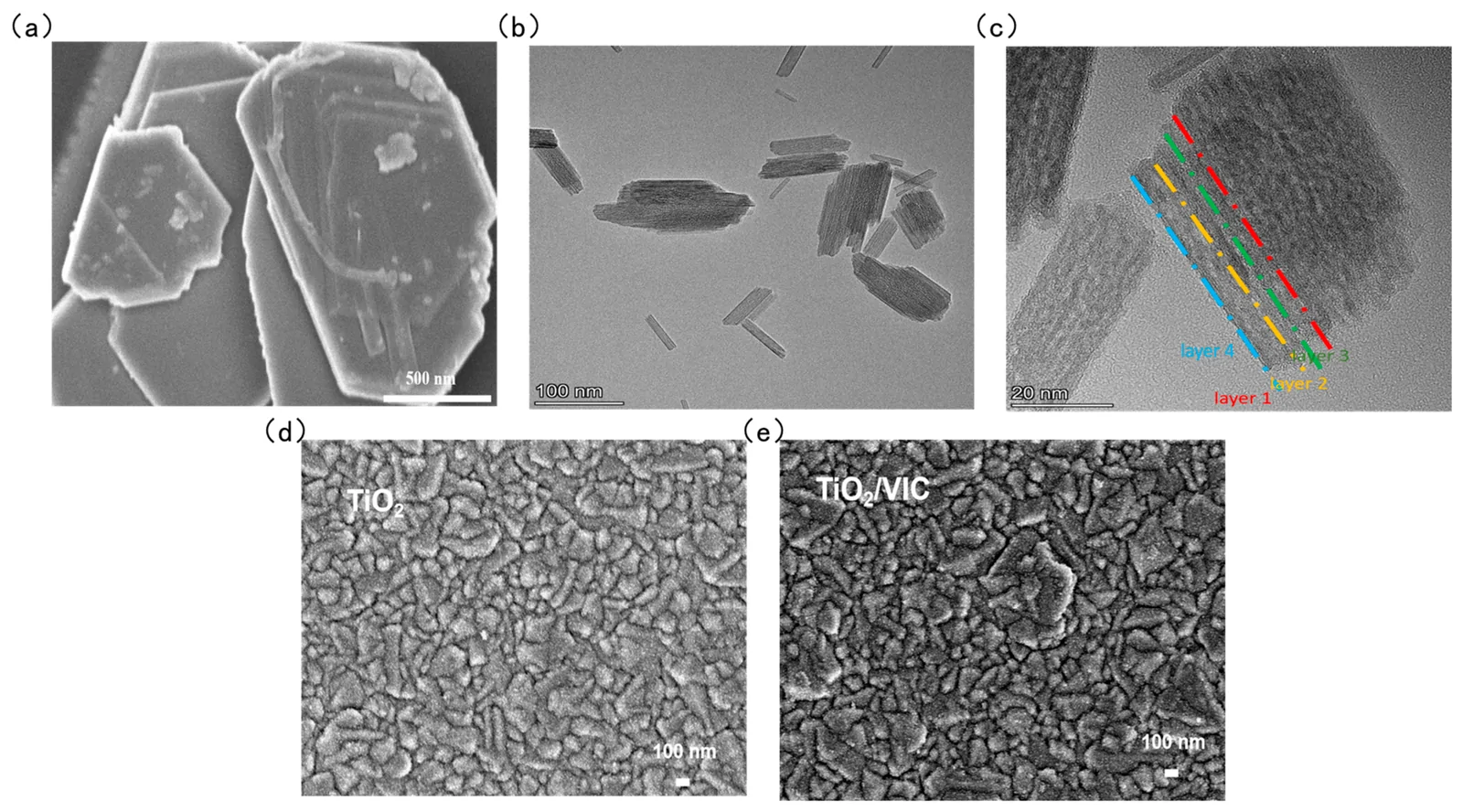
(**a**) SEM image of intercalated vermiculite; (**b**,**c**) TEM images of vermiculite nanosheets; (**d**) SEM image of the TiO_2_ film; (**e**) SEM image of the TiO_2_–vermiculite film.

**Figure 2:**
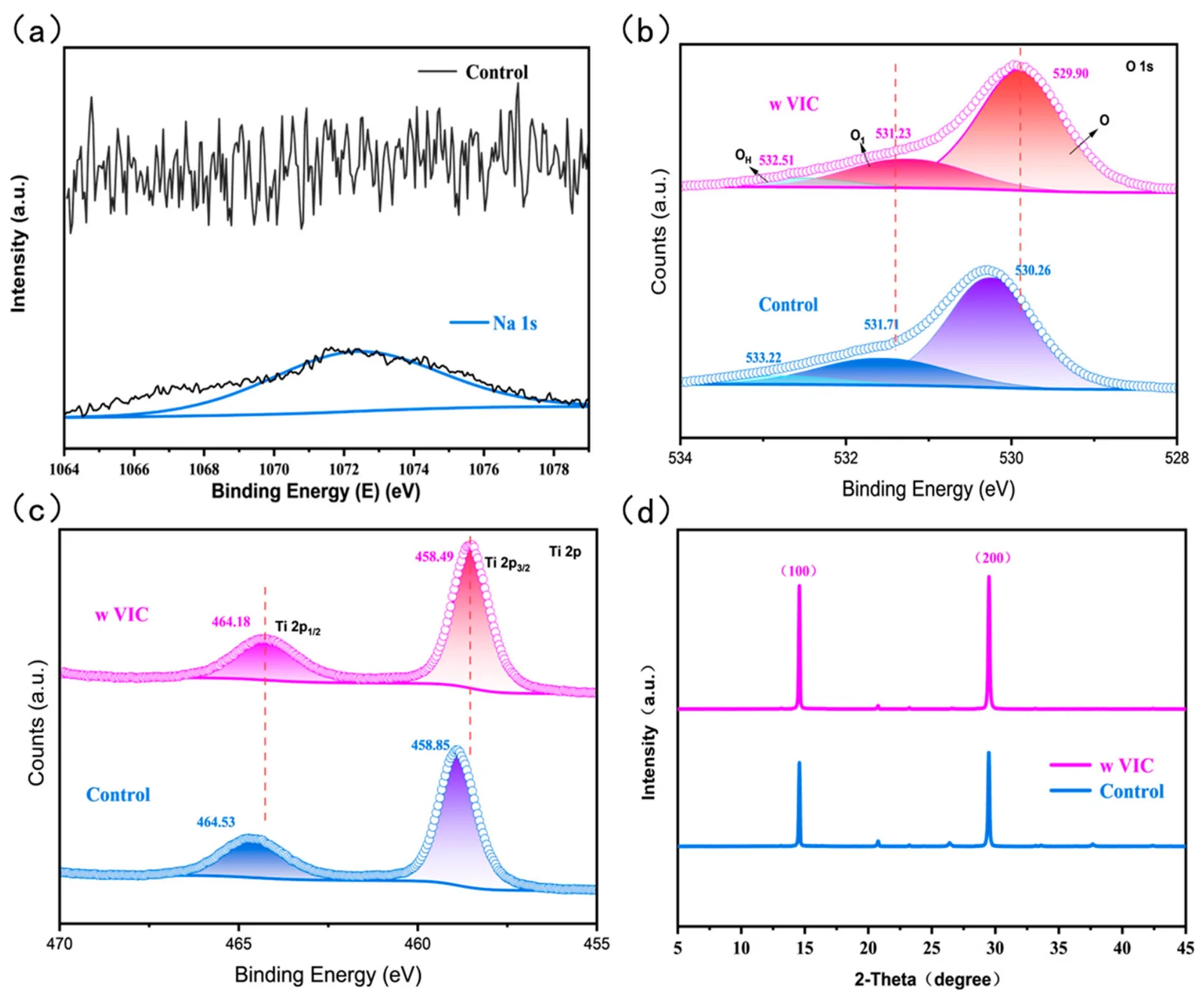
XPS spectra of TiO_2_ and TiO_2_–Na^+^-intercalated vermiculite films: (**a**) Na 1s, (**b**) Ti 2p, and (**c**) O 1s; (**d**) XRD patterns of the corresponding perovskite films.

**Figure 3:**
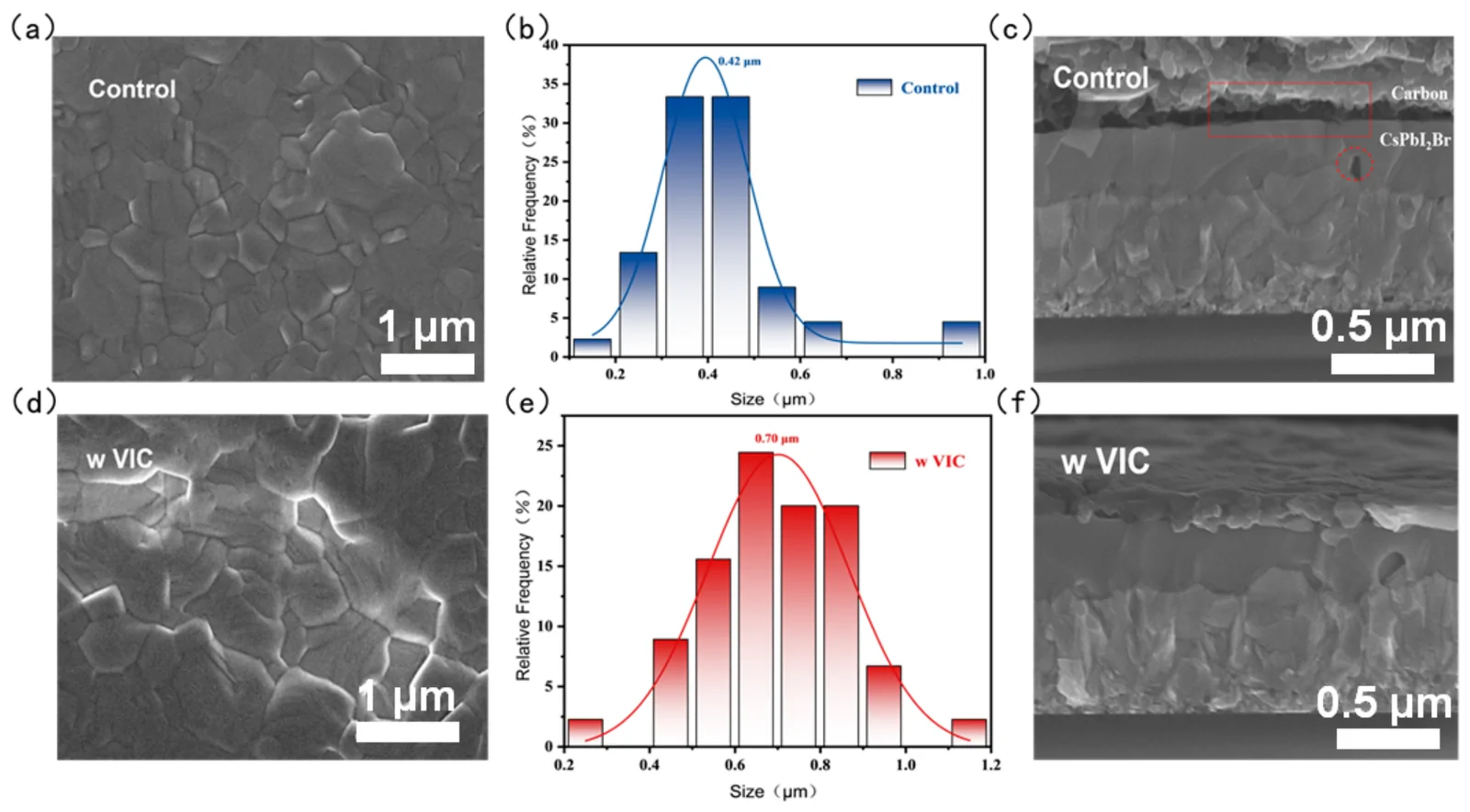
(**a**,**d**) Top-view SEM images, (**b**,**e**) grain-size distribution histograms, and (**c**,**f**) cross-sectional SEM images of CsPbI_2_Br films deposited on pristine and vermiculite-modified substrates.

**Figure 4:**
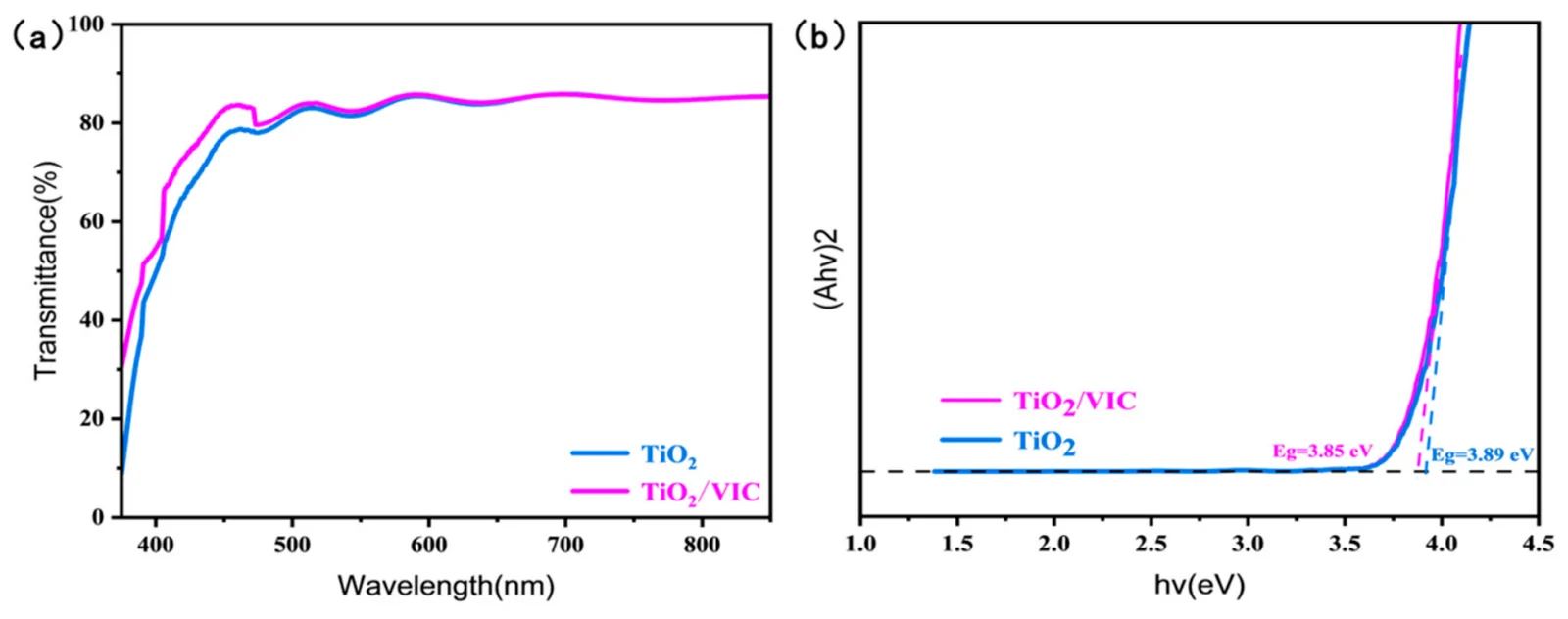
(**a**) UV–vis transmittance spectra and (**b**) Tauc plots of TiO_2_ and TiO_2_/vermiculite films.

**Figure 5:**
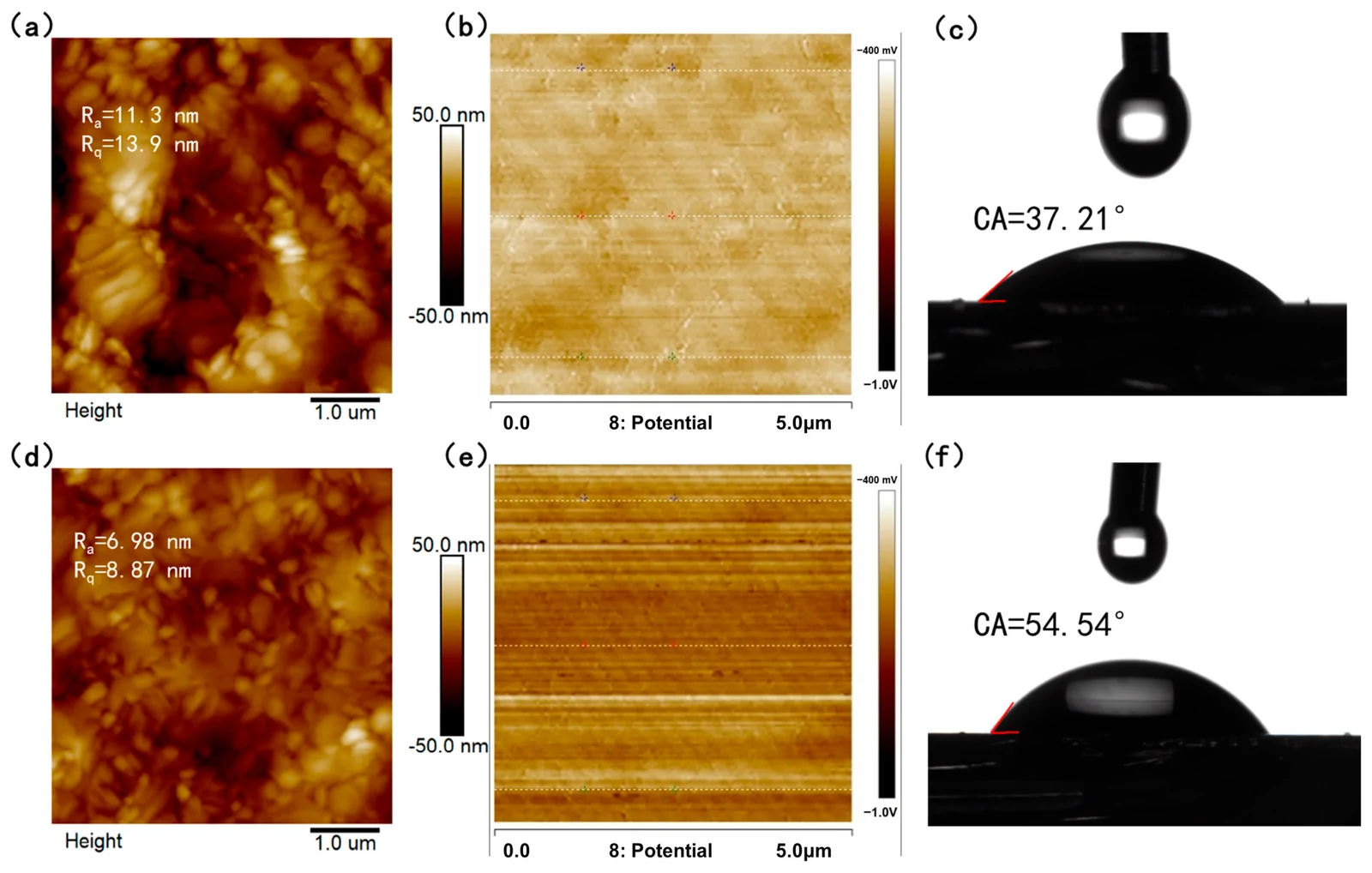
(**a**) AFM image, (**b**) KPFM image, and (**c**) water contact angle image of the control FTO/TiO_2_/CsPbI_2_Br film. (**d**) AFM image, (**e**) KPFM image, and (**f**) water contact angle image of the FTO/TiO_2_/vermiculite/CsPbI_2_Br film.

**Figure 6:**
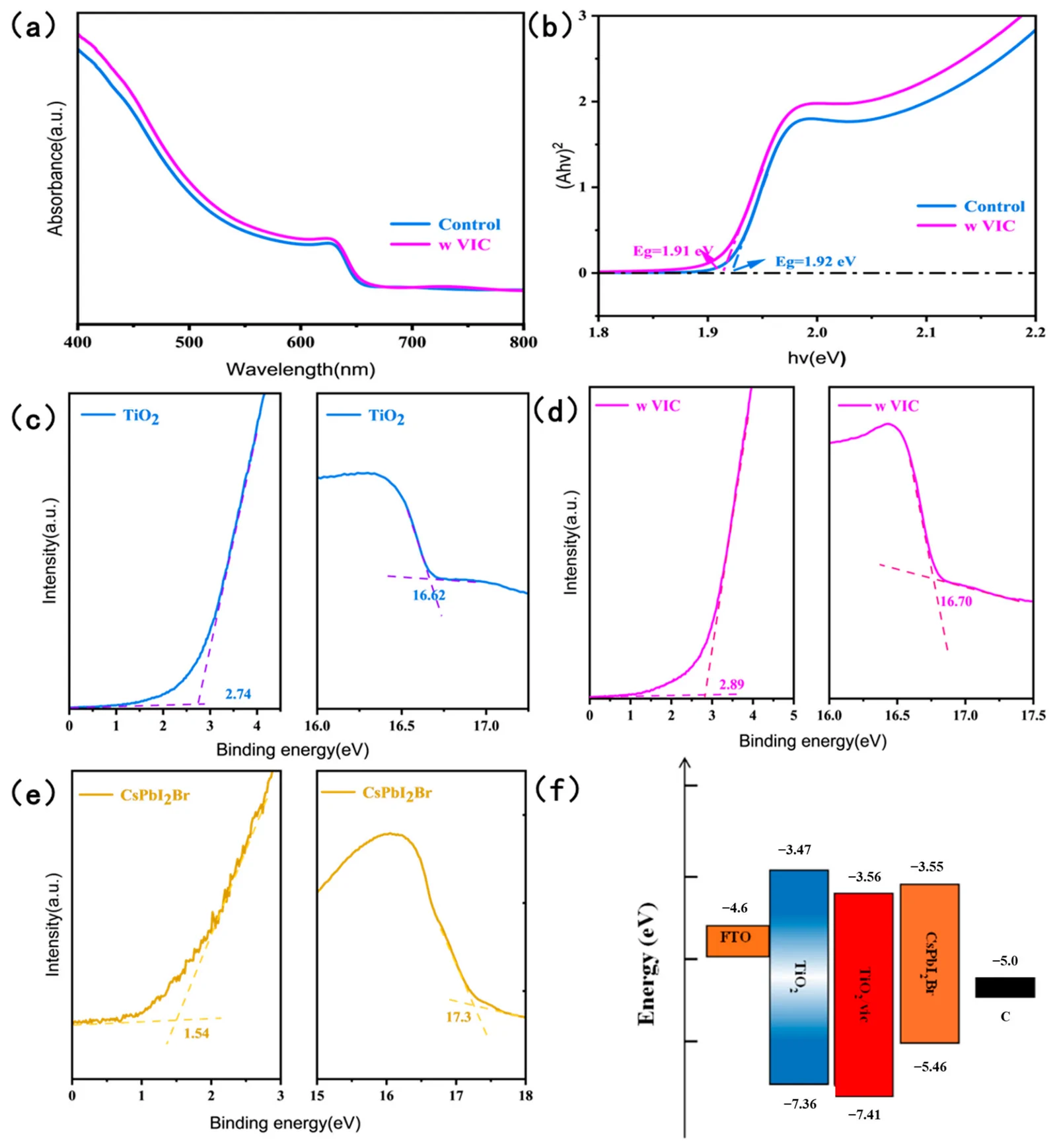
(**a**) UV–vis absorption spectra and (**b**) Tauc plots of TiO_2_/CsPbI_2_Br and TiO_2_/vermiculite/CsPbI_2_Br films; UPS spectra of (**c**) TiO_2_, (**d**) TiO_2_/vermiculite, and (**e**) CsPbI_2_Br films; (**f**) schematic energy-level alignment of the device.

**Figure 7:**
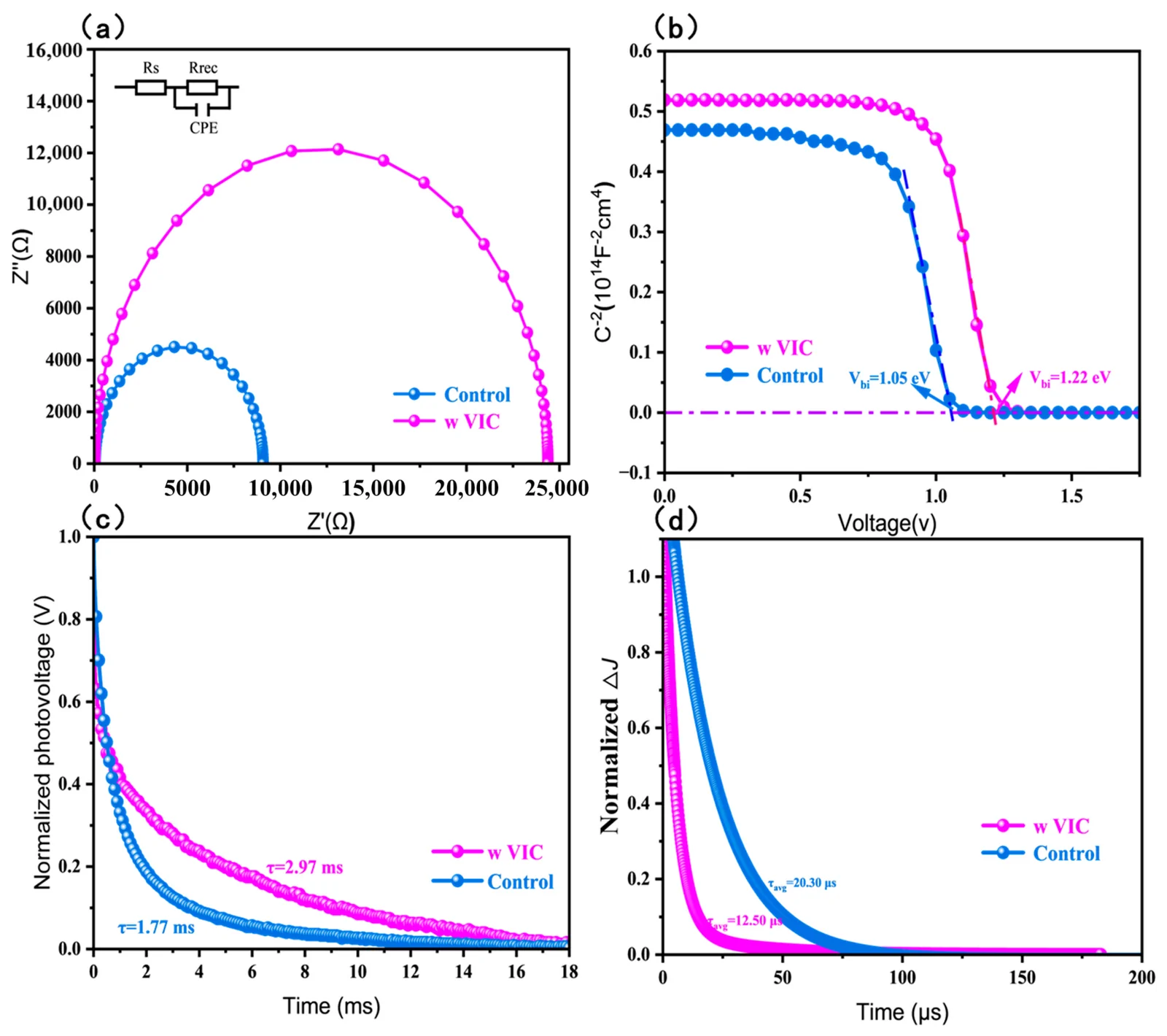
Electrical characterization of PSCs before and after vermiculite modification: (**a**) dark (without illumination) EIS Nyquist plots, (**b**) Mott–Schottky curves, (**c**) TPV curves, and (**d**) TPC curves.

**Figure 8:**
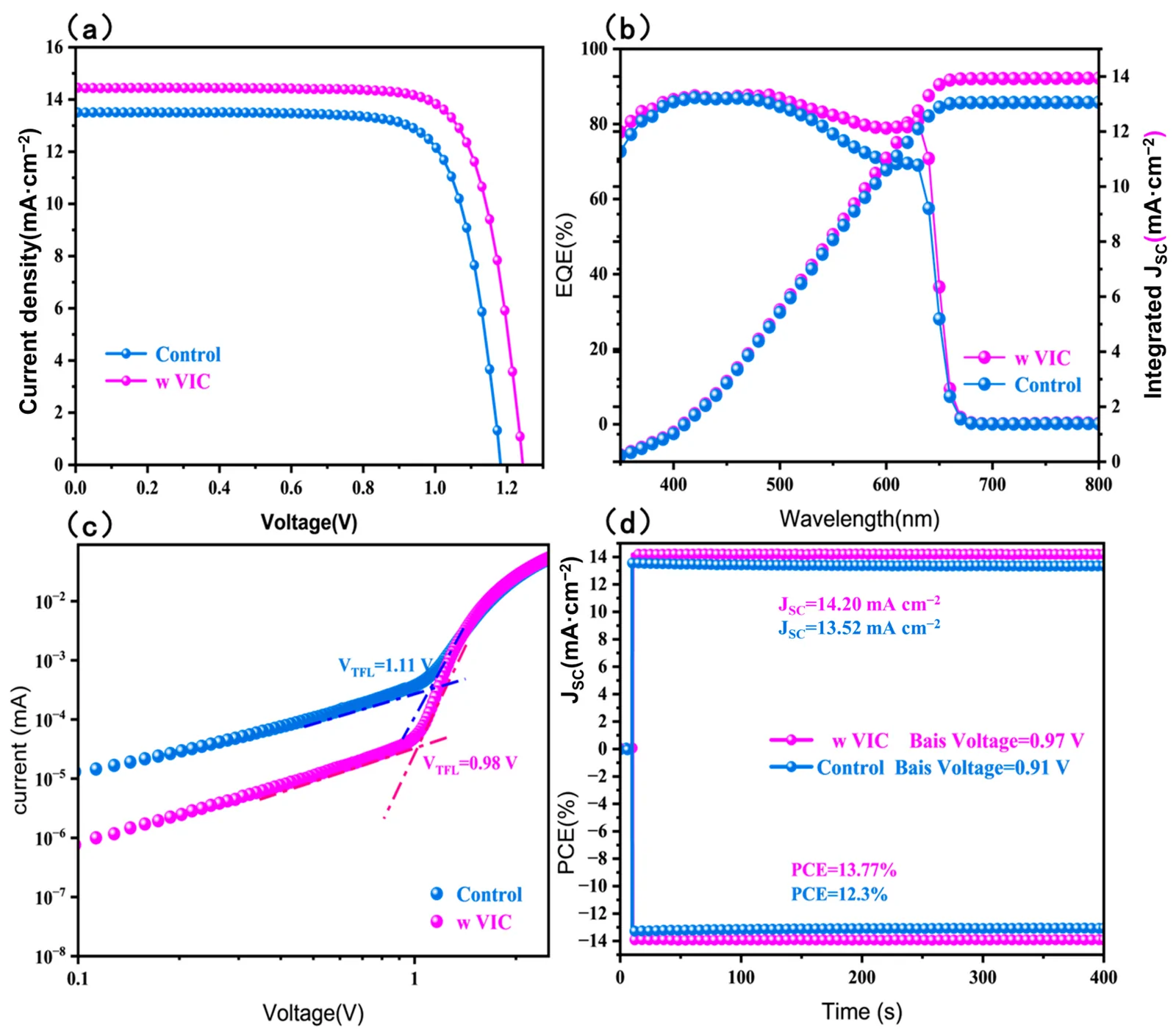
Device performance of PSCs before and after vermiculite modification: (**a**) J–V curves, (**b**) EQE spectra and integrated current densities, (**c**) SCLC curves, and (**d**) steady-state output at the maximum power point.

## Data Availability

The data presented in this study are available on request from the corresponding author due to intellectual property restrictions and a pending patent application.

## Supplementary Materials

The following supporting information can be downloaded at: https://www.mdpi.com/article/10.3390/nano16191216/s1.
