# Supplementary material for: Na+-Intercalated Two-Dimensional Vermiculite-Modified TiO2 Electron Transport Layer for Enhanced CsPbI2Br Perovskite Solar Cells
Source: Nanomaterials (Basel). 2026 Sep 26;16(19):1216. doi: 10.3390/nano16191216 (PMC13667137; doi:10.3390/nano16191216)
Supplement: Supplementary file 1 [file nanomaterials-16-01216-s001.zip › nanomaterials-4579110-supplementary.pdf]

Supporting Information

Article

Na^{+}-Intercalated Two-Dimensional Vermiculite-Modified TiO_2 Electron Transport Layer for Enhanced $CsPbI_2Br$ Perovskite Solar Cells

Yuan Xu [1,2,*,†], Yaxuan Chai [3,†], Kaituo Zhang [2,*], Fengli Liu [3], Tiantian Li [4], Ke Xu [2], Xian Hua [4], Pengju Guo [1], Fachuang Li [1] and Lan Zhang [3,*]

1 Xinxiang Engineering Research Center for Advanced Energy Storage Batteries and Materials, School of Materials Science and Engineering, Henan Institute of Technology, Xinxiang 453003, China
2 Henan Key Laboratory of Advanced Cable Materials and Intelligent Manufacturing, School of Cable Engineering, Henan Institute of Technology, Xinxiang 453003, China
3 Fujian Key Laboratory of Photoelectric Functional Materials, College of Materials Science & Engineering, Huaqiao University, Xiamen 361021, China
4 Engineering Technology Education Center, Henan Institute of Technology, Xinxiang 453003, China
* Correspondence: xuyuan94@163.com (Y.X.); zkt8009@163.com (K.Z.); lanzhang@hqu.edu.cn (L.Z.)
† These authors contributed equally to this work.

Supplementary Experimental Details

S1. Materials and Device Fabrication Details

S1.1. Materials

Fluorine-doped tin oxide (FTO) glass substrates (14 Ω sq^{-1}), Lead iodide (PbI_2, 99.99%), lead bromide ($PbBr_2$, 99.99%) and cesium iodide (CsI, 99.99%) were purchased from Liaoning Youxuan New Energy Technology Co., Ltd. Titanium tetrachloride ($TiCl_4$, 99%) was obtained from Macklin. Dimethyl sulfoxide (DMSO, 99.7%), N,N-dimethylformamide (DMF, 99.5%), isopropanol, acetonitrile, and absolute ethanol were used as received. Conductive carbon paste was obtained from Guangzhou Seaside Technology Co., Ltd. Natural vermiculite and NaCl were also used as received.

S1.2. FTO cleaning and patterning

FTO glass was patterned by laser etching before film deposition. The etched region was approximately 3 mm × 15 mm. The substrates were cleaned with detergent and deionized water, and then ultrasonically cleaned sequentially in deionized water and absolute ethanol. After drying at 60 °C, the substrates were used for subsequent deposition.

S1.3. TiO_2 compact layer preparation

A $TiCl_4$ aqueous precursor solution was prepared by slowly adding 4.5 mL $TiCl_4$ into 200 mL ice-cooled deionized water under continuous stirring. The cleaned FTO substrates were treated with UV–ozone for 30 min and immersed in 40 mL of the $TiCl_4$ solution at 70 °C for approximately 24 min. After deposition, the substrates were rinsed thoroughly with deionized water and ethanol, dried, and annealed at 200 °C for 1 h in air.

S1.4. Purification and dispersion of Na^+-treated vermiculite nanosheets

Natural vermiculite was treated in 3.4 M NaCl aqueous solution at 180 °C for 12 h. After the reaction, the product was collected by centrifugation and washed repeatedly with deionized water to remove residual soluble NaCl. Each washing step involved redispersing the collected solid in deionized water followed by centrifugation. This washing/redispersion/centrifugation process was repeated multiple times to minimize residual soluble salts in the supernatant before the final exfoliation step. The purified product was then redispersed in deionized water and exfoliated by ultrasonication. This purification process was intended to minimize residual soluble NaCl before film deposition. The supernatant containing exfoliated nanosheets was collected and filtered to remove unexfoliated large

particles, yielding a stable vermiculite nanosheet dispersion for TiO_2 modification. The concentration of the collected dispersion was 20 mg mL^{-1}.

S1.5. Perovskite precursor and film deposition

The $CsPbI_2Br$ precursor solution was prepared by dissolving 311.8 mg CsI, 276.6 mg PbI_2, and 220.2 mg $PbBr_2$ in 1 mL DMSO in a nitrogen-filled glovebox. The precursor concentration was 1.2 M based on the $CsPbI_2Br$ formula unit. The solution was filtered through a 0.22 μm organic filter before use.

Prior to perovskite deposition, the TiO_2-coated substrates were treated with UV–ozone for 15 min and plasma cleaning for 5 min, followed by preheating at 60 °C for 10 min. A volume of 50 μL precursor solution was spin-coated using a two-step process (700 rpm for 5 s, then 3500 rpm for 35 s). No antisolvent was used during spin coating. The precursor films were first heated at 38 °C for 80 s and subsequently annealed at 180 °C for 10 min. To evaluate whether residual soluble ionic species could account for the observed effects, a NaCl-only control was prepared without vermiculite nanosheets. For the NaCl-only control, an aqueous NaCl solution (10 mM) was spin-coated onto the TiO_2 substrate using the same dispensing volume, spin-coating program, and post-annealing conditions as those used for the vermiculite-modification step. The 10 mM NaCl treatment was used as a representative NaCl-only control rather than a quantitatively matched comparison. All subsequent perovskite deposition and carbon-electrode fabrication procedures were identical to those used for the standard devices.

S1.6. Carbon electrode fabrication

The exposed contact region was defined by wiping the edge of the conductive substrate with a cotton swab lightly wetted with DMF. The uncovered strip was approximately 1 mm×15 mm. Commercial conductive carbon paste was deposited by screen printing and annealed at 120 °C for 15 min.

S1.7. Device area and statistics

The active area of the photovoltaic devices was 0.04 cm^2. A metal shadow mask with the same aperture area was used during J-V testing. Unless otherwise noted, the photovoltaic statistics were collected from at least 20 devices fabricated from 3 independent batches.

S2. Photovoltaic Measurement Details

S2.1. J–V measurements

The current density-voltage (J-V) characteristics were measured under simulated AM 1.5G illumination (100 mW cm^{-2}) using a Newport solar simulator and a PVIV-94043A test system. The light intensity was calibrated using a certified silicon reference cell equipped with a KG-2 filter before measurement. The devices were measured using a metal shadow mask defining an active area of 0.04 cm^2.

Unless otherwise specified, the J-V curves shown in the manuscript were recorded in the reverse scan direction from 1.5 V to 0 V at a scan rate of 100 mV s^{-1}. The voltage delay time was set to 10 ms. The photovoltaic parameters were extracted directly from the measured J-V curves.

S2.2. EQE measurements

External quantum efficiency (EQE) spectra were recorded using a QE-R quantum efficiency measurement system (Enli Technology Co., Ltd.). The spectra were collected over the wavelength range of 300–800 nm. The integrated current density from the EQE spectra was compared with the JSC values obtained from J–V measurements.

S3. Electrical and Electrochemical Characterization

S3.1. Electrochemical impedance spectroscopy (EIS)

EIS measurements were carried out using a Zahner IM6ex electrochemical workstation. The spectra were recorded in the dark at 0 V bias over a frequency range from 1 MHz to 1 Hz with an AC perturbation amplitude of 10 mV. The measured Nyquist plots were fitted using an equivalent circuit model to extract the series resistance (Rs) and recombination resistance (Rrec).

S3.2. Mott–Schottky measurements

Mott-Schottky measurements were performed on a Zahner IM6ex electrochemical workstation under dark conditions at a frequency of 10 kHz with an AC amplitude of 10 mV. The capacitance–voltage relationship was analyzed according to the Mott–Schottky equation:

$$\frac{1}{C^2}=\frac{2}{A^2 q\varepsilon\varepsilon_0 N_D}(V_{bi}\text{-}V)$$

Where ε_0 is the vacuum dielectric constant, ε is the dielectric constant of the perovskite film, which is generally taken as 12, and L is the thickness of the film, which is generally taken as 400 nm (measured from cross-sectional SEM).

S3.3. TPV and TPC measurements

Transient photovoltage (TPV) and transient photocurrent (TPC) measurements were performed using a Zahner IM6ex electrochemical workstation under pulsed light perturbation superimposed on a steady background illumination. TPV was measured under open-circuit condition, while TPC was measured under short-circuit condition. The decay curves were used to evaluate carrier recombination and charge extraction dynamics.

S3.4. SCLC measurements

The trap-state density was estimated by the space-charge-limited current (SCLC) method from dark current–voltage curves. The trap-state density Nt was calculated according to:

$$N_t = \frac{2\varepsilon_0 \varepsilon V_{TFL}}{qL^2}$$

Where ε_0 is the vacuum dielectric constant, ε is the relative dielectric constant of the perovskite (taken as 12), and L is the thickness of the film (taken as 400 nm).

S3.5. Light-intensity-dependent measurements

The dependence of V_{OC} and J_{SC} on light intensity was measured under different illumination intensities by adjusting the output of the solar simulator. The ideality factor n was estimated from the slope of the V_{OC} versus ln(light intensity) plot according to:

$$V_{OC} = \frac{nKT}{e} \ln\left(\frac{J_{SC}}{J_0} + 1\right)$$

e is the basic charge, *K* is Boltzmann constant, *T* is the temperature. The bimolecular recombination is existed when *n* is close to 1, and the Shockley-Read-Hall (SRH) recombination is occurred when *n* is close to 2.

S4. Structural, Surface, and Spectroscopic Characterization

S4.1. SEM and XRD

Scanning electron microscopy (SEM) measurements were performed using a Hitachi SU8010 field-emission scanning electron microscope. X-ray diffraction (XRD) patterns were recorded on a Rigaku SmartLab diffractometer using Cu Kα radiation (λ=0.15418 nm).

S4.2. AFM and KPFM

Atomic force microscopy (AFM) and Kelvin probe force microscopy (KPFM) measurements were carried out using an MFP-3D atomic force microscope (Oxford Instruments). The measurements were performed in tapping mode under ambient conditions. Typical scan areas were 5 μm×5 μm. The surface potential maps were obtained simultaneously with the topography images to evaluate local work-function variations.

S4.3. XPS and UPS

X-ray photoelectron spectroscopy (XPS) and ultraviolet photoelectron spectroscopy (UPS) measurements were performed on a Thermo Scientific K-Alpha+ photoelectron spectrometer. For XPS measurements, charge compensation was applied using the instrument's built-in neutralization system, and the binding energies were calibrated using the adventitious C 1s peak at 284.8 eV. UPS spectra were recorded using He I radiation (21.22 eV). The valence-band maximum and work function were estimated from the onset and cutoff regions of the UPS spectra.

S4.4. UV-vis absorption

UV-vis absorption spectra were collected using a Lambda 950 UV/Vis/NIR spectrophotometer in the wavelength range of 200–900 nm. The optical bandgap values were estimated from Tauc plots.

S4.5. Steady-state PL and Time-resolved PL

The PL and TRPL measurements were performed on perovskite films deposited on FTO-based substrates, namely FTO/TiO_2/$CsPbI_2Br$ and FTO/TiO_2/vermiculite/$CsPbI_2Br$. Steady-state photoluminescence (PL) spectra were characterized by Thermo Scientific Lumina (LF-1303003). An Omni-λ Monochromator/ Spectrograph (Zolix) with the time-correlated single-photon counting method (PicoHarp 300) was used to record the TRPL spectra, and the key parameters are extracted by fitting with a biexponential rate law:

$$f(t)=\Sigma A_i \, exp^{\frac{\tau}{\tau^i}} (i=1,2) \qquad \text{(S1)}$$

A is the weighting factor, and t is the weighting constant. The τ_1 and τ_2 are used to represent the fast

decay component and the slow decay component, respectively. The fast decay component (τ_1) is commonly associated with faster interfacial/surface-related decay processes, whereas the slow decay component (τ_2) is generally attributed to slower bulk-related recombination processes. These assignments are phenomenological and are used here mainly for comparative analysis. The τ_{ave} can be calculated by the formula:

$$\tau_{ave}=\frac{\Sigma A_i \tau_i^2}{\Sigma A_i \tau_i} \ (i=1,2) \qquad \text{(S2)}$$

The τ_{ave} can be used to estimate the average carrier lifetime. The smaller the τ_{ave} value, the shorter the residence time of the film carriers from the excited state to the quenched state that is more conducive to the transport and extraction of holes.

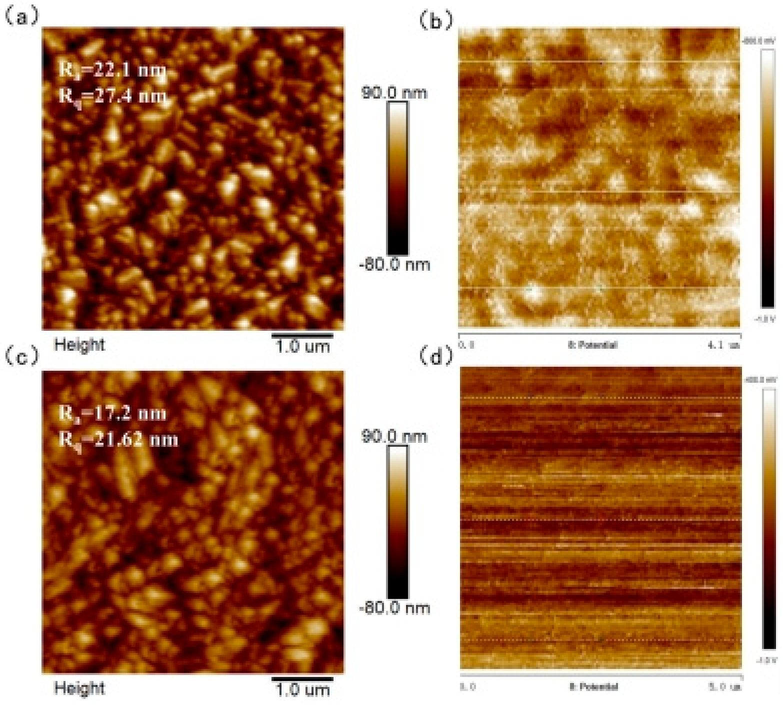

Figure S1 (a) AFM image and (b) KPFM image of the control FTO/TiO_2 film; (c) AFM image and (d) KPFM image of the FTO/TiO_2/vermiculite film.

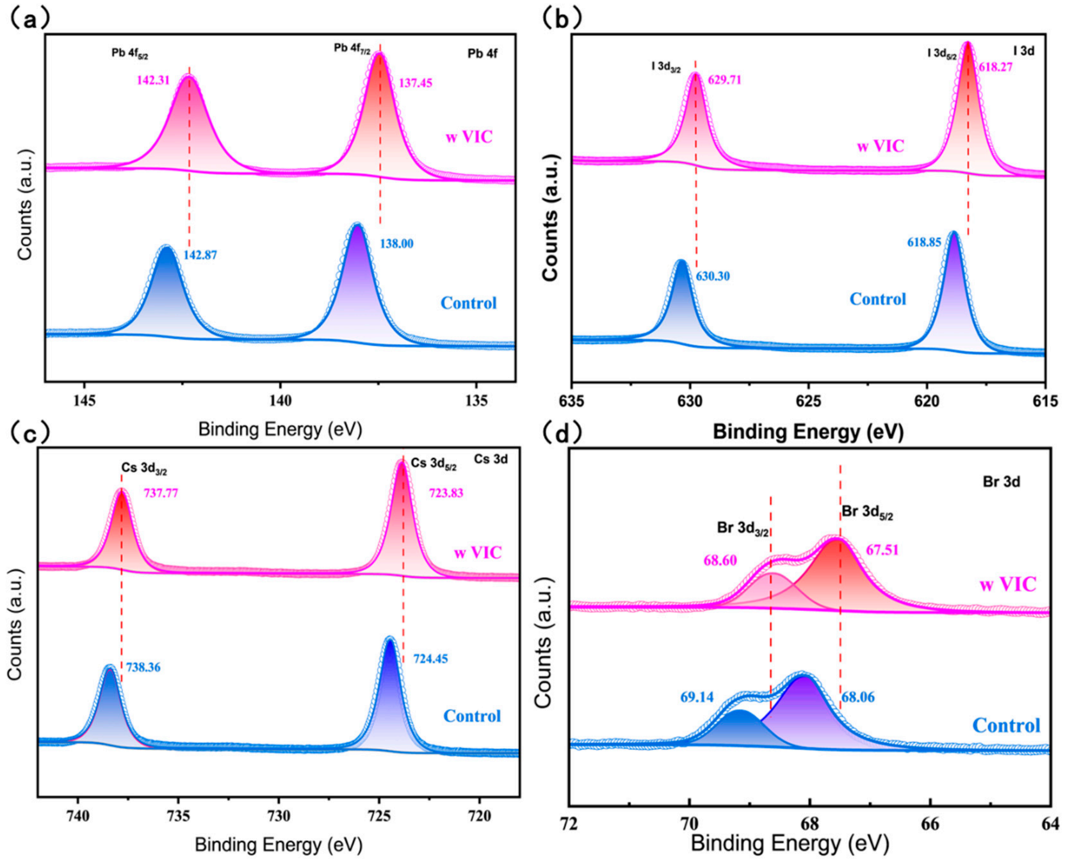

Figure S2. XPS spectra of $CsPbI_2Br$ films before and after vermiculite modification: (a) Pb 4f, (b) I 3d, (c) Cs 3d, and (d) Br 3d.

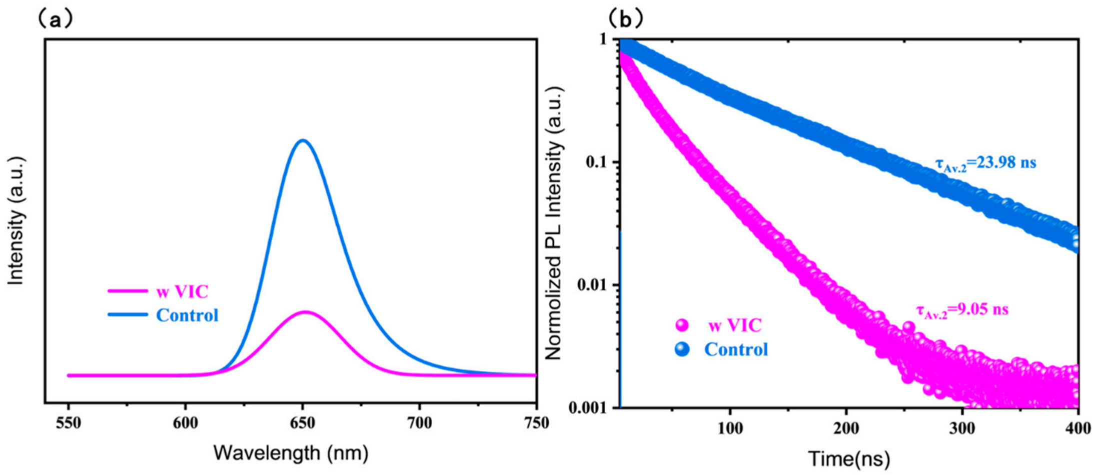

Figure S3. (a) Steady-state PL spectra and (b) TRPL spectra of $FTO/TiO_2/CsPbI_2Br$ and FTO/TiO_2/vermiculite/$CsPbI_2Br$ films.

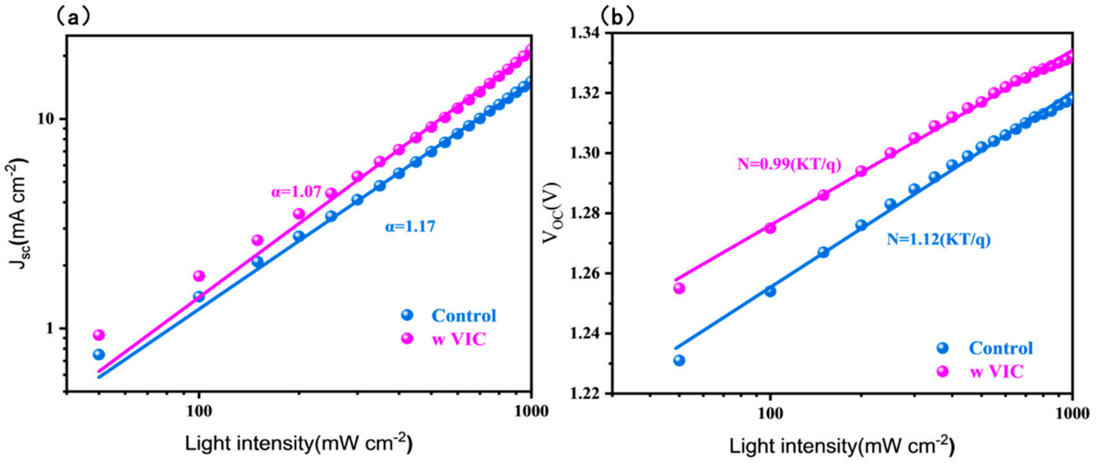

Figure S4 Light-intensity dependence of the device parameters: (a) JSC versus light intensity and (b) VOC versus light intensity.

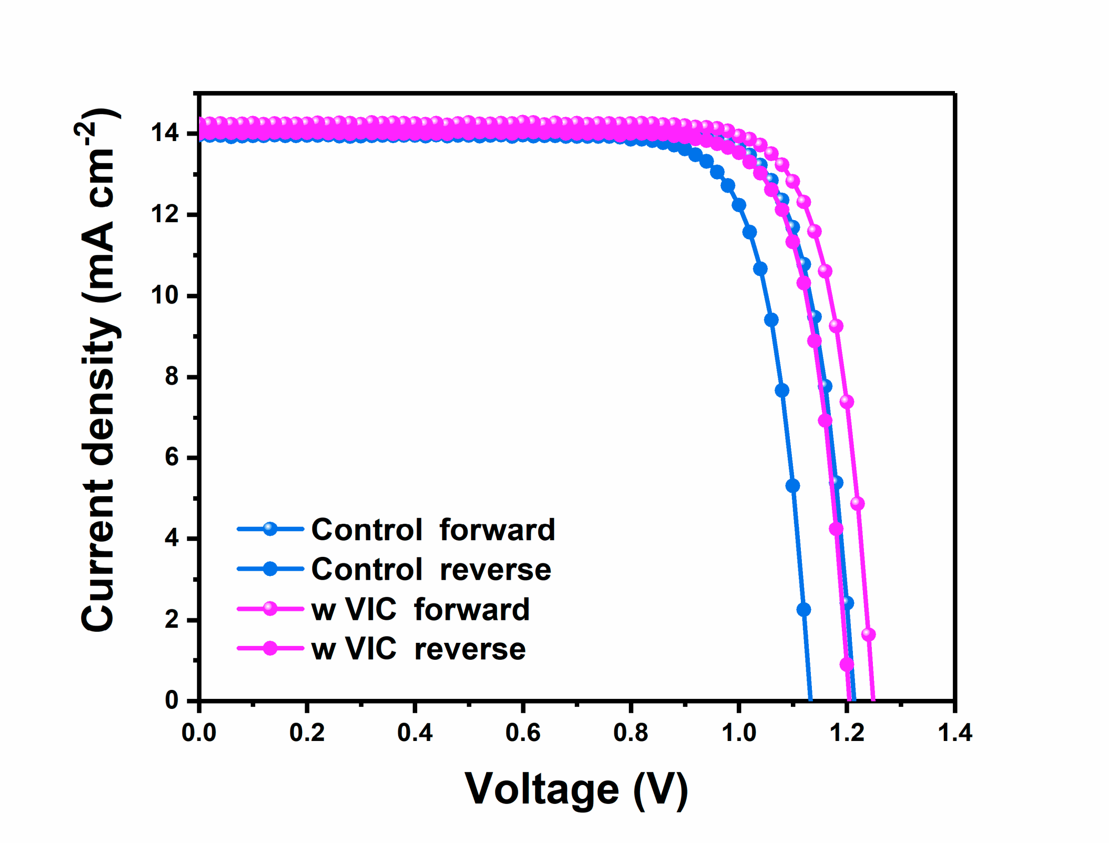

Figure S5 Forward/reverse J–V curves and hysteresis index of control and vermiculite-modified devices.

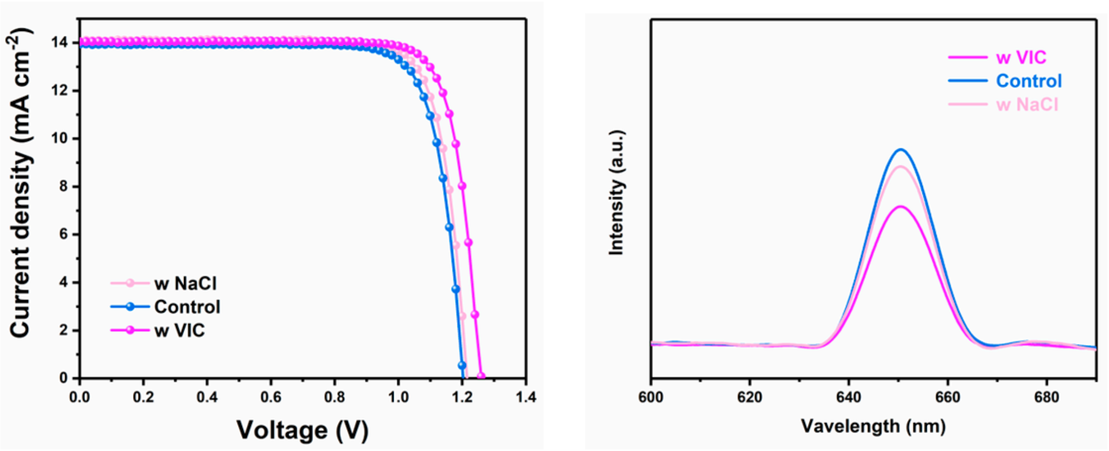

(a) (b)

Figure S6 Comparison of (a) J–V characteristics and (b) steady-state PL spectra for the control sample without interfacial modifier, the NaCl-only control, and the VIC-modified sample..

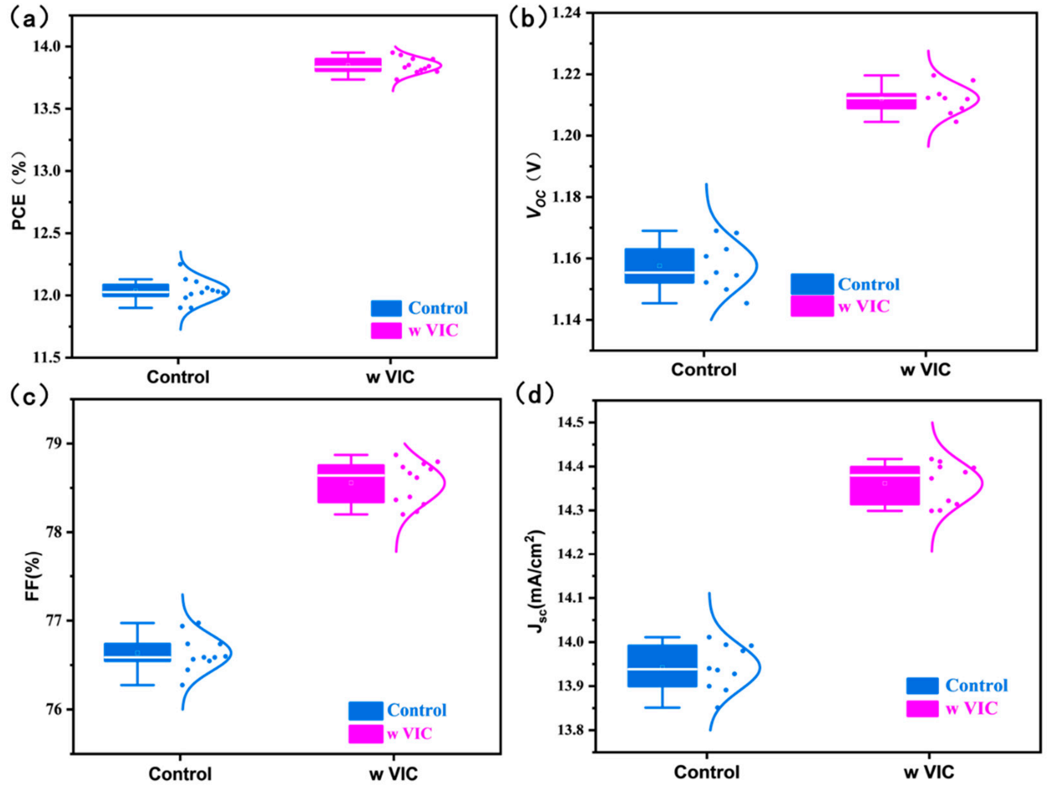

Figure S7 Box plots of device parameters for PSCs before and after vermiculite modification: (a) PCE, (b) V_{OC}, (c) FF, and (d) J_{SC}.

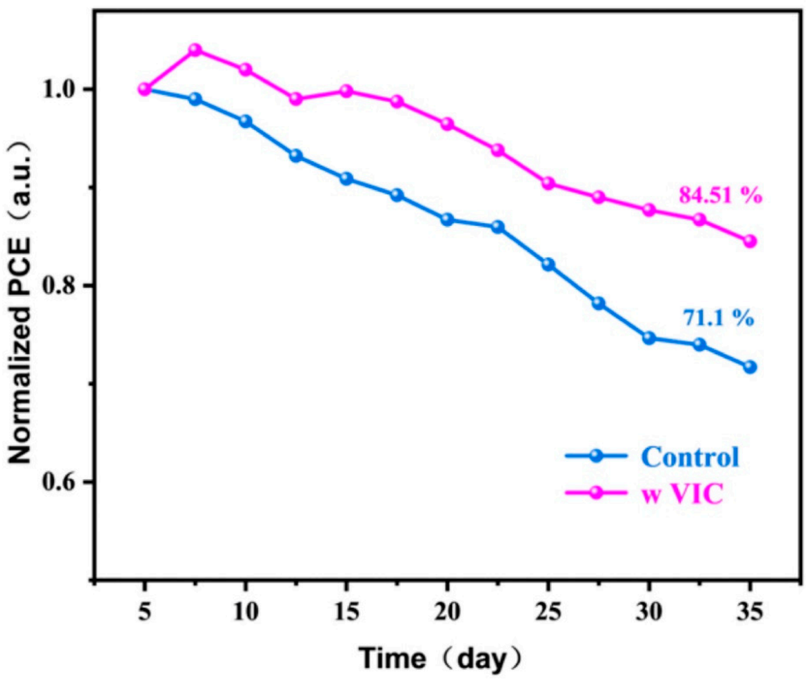

Figure S8 Storage stability of the devices under ambient conditions at room temperature (RH = 25 ± 10%).

Table S1 Comparison between the present work and the previously reported alkali-intercalated vermiculite-based PSCs

Reference	Architecture	Perovskite	Modifier	Intercalated ion	Main emphasis	PCE	Distinction
Ref. [30]	Carbon-based PSC	$CsPbI_2Br$ / related	Pre-buried interface	multiple alkali ions	templating growth / ionic regulation	15.25%	broader alkali-ion screening
This work	FTO/TiO_2/perovskite/carbon	$CsPbI_2Br$	TiO_2-side buried interface	Na+	TiO_2 surface smoothing, KPFM surface potential, UPS energy-level alignment, Ti 2p/O 1s changes, charge extraction	13.94%	focused ETL-side interfacial regulation study

Table S2. TRPL fitting parameters of $CsPbI_2Br$ films based on TiO_2 and TiO_2/vermiculite ETLs.

Sample	A_1（%）	τ_1（ns）	A_2（%）	τ_2（ns）	τ_{ave}（ns）
Control	19.03	7.916	80.97	27.765	23.98
w VIC	56.47	12.466	43.53	4.618	9.05

Table S3. Energy-level parameters of TiO_2, TiO_2/vermiculite, and $CsPbI_2Br$ films.

Sample	E_{onset} （eV）	E_{Cutoff} （eV）	E_g （eV）	E_F （eV）	VBM （eV）	CBM （eV）
TiO_2	2.76	16.62	3.89	4.6	−7.36	−3.47
TiO_2/VIC	2.89	16.70	3.85	4.52	−7.41	−3.56
$CsPbI_2Br$	1.54	17.30	1.91	3.92	−5.46	−3.55

Table S4 Fitted EIS parameters of PSCs based on TiO_2 and TiO_2/vermiculite ETLs.

Sample	R_s（Ω）	R_{rec}（Ω）	CPE
Control	51.28	9023	9.356E-9
VIC-modified	30.05	22476	1.053E-8

Table S5 Photovoltaic parameters of PSCs before and after vermiculite modification.

PSC	V_{OC}（V）	J_{SC}（mA cm^{-2}）	FF（%）	PCE（%）
Control	1.18	13.50	76.80	12.25
VIC-modified	1.24	14.44	77.60	13.94

Table S6 Photovoltaic parameters of PSCs before and after vermiculite modification under forward and reverse scans.

PSC	Scan direction	V_{OC} （V）	J_{SC} （mA cm^{-2}）	FF （%）	PCE （%）	HI
Control	reverse	1.22	14.13	76.42	13.17	0.11
	forward	1.13	13.96	74.32	11.72	
VIC-modified	reverse	1.25	14.18	77.51	13.74	0.06
	forward	1.21	13.96	76.24	12.88	
